# Assay establishment and validation of a high-throughput organoid-based drug screening platform

**DOI:** 10.1186/s13287-022-02902-3

**Published:** 2022-05-26

**Authors:** Xiaomeng Li, Guoxiang Fu, Long Zhang, Ruoyu Guan, Peiyuan Tang, Jialing Zhang, Xinxin Rao, Shengzhi Chen, Xiaoya Xu, Yi Zhou, Yun Deng, Tao Lv, Xingfeng He, Shaobo Mo, Peiyuan Mu, Jianjun Gao, Guoqiang Hua

**Affiliations:** 1 Institute of Radiation Medicine, Shanghai Medical College, Fudan University, Shanghai, 200032 China; 2 D1 Medical Technology Company, Shanghai, 201802 China; 3 Department of Colorectal Surgery, Fudan University Shanghai Cancer Center, Fudan University, Shanghai, 200032 China; 4 Department of Oncology, Shanghai Medical College, Fudan University, Shanghai, 200032 China; 5 Department of Radiation Oncology, Fudan University Shanghai Cancer Center, Fudan University, Shanghai, 200032 China; 6 Cancer Institute, Fudan University Shanghai Cancer Center, Fudan University, Shanghai, 200032 China

**Keywords:** Organoid, Z-stack, Fluorescence, High-throughput, Drug screening

## Abstract

**Background:**

Organoids are three-dimensional structures that closely recapitulate tissue architecture and cellular composition, thereby holding great promise for organoid-based drug screening. Although growing in three-dimensional provides the possibility for organoids to recapitulate main features of corresponding tissues, it makes it incommodious for imaging organoids in two-dimensional and identifying surviving organoids from surrounding dead cells after organoids being treated by irradiation or chemotherapy. Therefore, significant work remains to establish high-quality controls to standardize organoid analyses and make organoid models more reproducible.

**Methods:**

In this study, the Z-stack imaging technique was used for the imaging of three-dimensional organoids to gather all the organoids’ maximum cross sections in one imaging. The combination of live cell staining fluorescent dye Calcein-AM and ImageJ assessment was used to analyze the survival of organoids treated by irradiation or chemotherapy.

**Results:**

We have established a novel quantitative high-throughput imaging assay that harnesses the scalability of organoid cultures. Using this assay, we can capture organoid growth over time, measure multiple whole-well organoid readouts, and show the different responses to drug treatments.

**Conclusions:**

In summary, combining the Z-stack imaging technique and fluorescent labeling methods, we established an assay for the imaging and analysis of three-dimensional organoids. Our data demonstrated the feasibility of using organoid-based platforms for high-throughput drug screening assays.

**Graphical Abstract:**

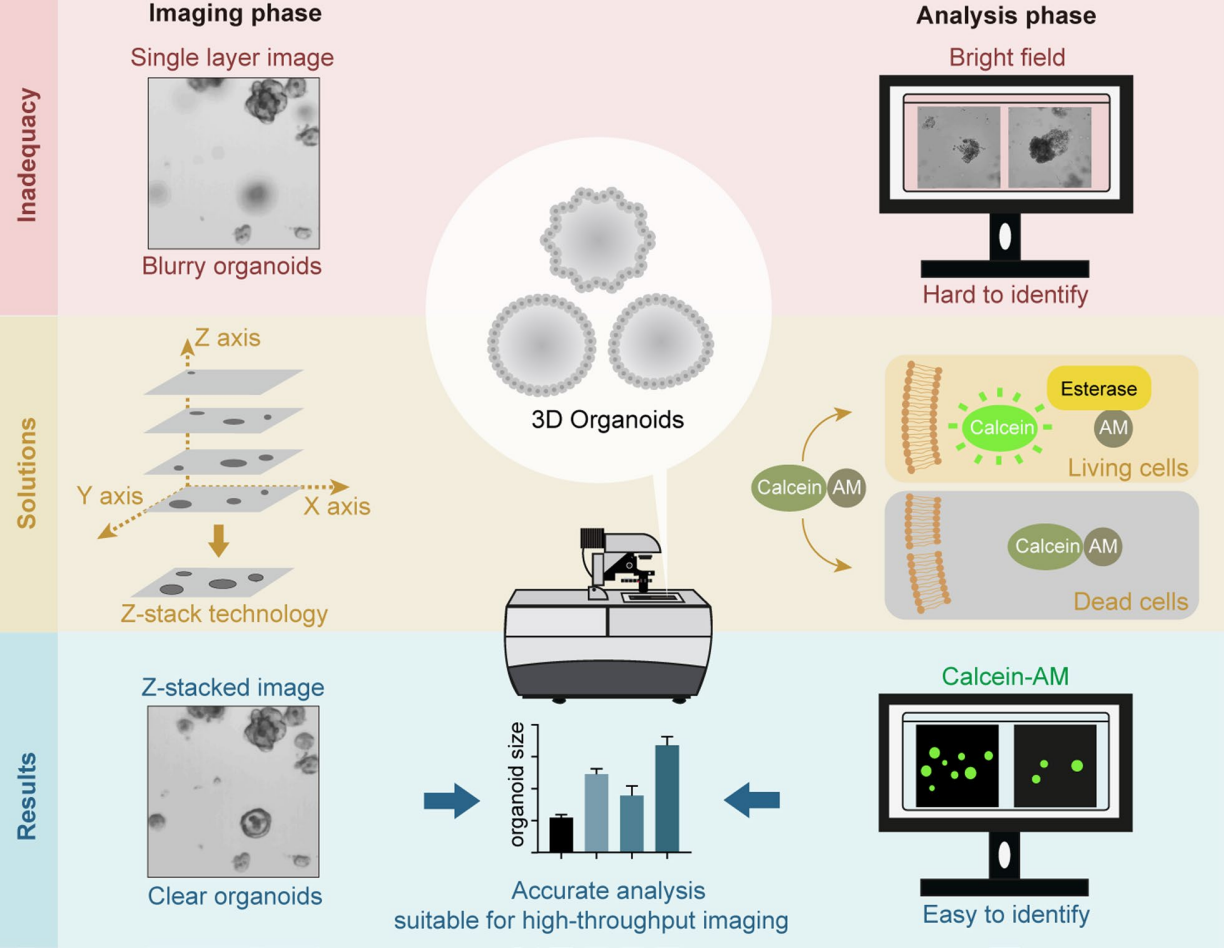

**Supplementary Information:**

The online version contains supplementary material available at 10.1186/s13287-022-02902-3.

## Background

Lacking three-dimensional (3D) cell-to-cell contacts and the interactions between cells and extracellular supporting matrix, two-dimensional (2D) cultured cells fail to mimic the physiological microenvironment of in vivo organs [1, 2]. Due to the discrepancy between the simplicity of cell models and the complexity of human organs, the consistency of drug safety and efficacy between basic studies using cell lines and preclinical trials is poor. A great deal of promising “hits” have failed in animal models and human clinical trials, causing dramatic increases in the cost of drug discovery [3–5]. In recent years, stem-cell-derived organoids have been thought to be promising tools for drug screening and pharmacological research, which could bridge the gap between 2D cell lines and living organs [4]. Organoids are known as 3D cultured multicellular organ-like aggregates, which are also called micro-organs. Compared with 2D cell lines, organoids have the potential to mimic cell niches more realistically [2, 6]. Therefore, organoids are recognized as promising models for the development of next-generation drug screening platforms [7, 8]. Many studies have validated that patient-derived organoids (PDOs) could predict the patients’ responses to clinical treatments [9–12]. PDOs show great applicable prospects in personalized medicine and drug development [13]. Intestinal organoids are easy to handle and their culture systems are relatively mature, which is expected to pave the way for organoids in drug screening and precision medicine [14, 15].

Although there are promising prospects for organoids in drug development and precision medicine, some issues need to be solved before their large-scale applications. First, although Matrigel provides a 3D microenvironment for organoids to grow [16], limited by imaging technology, it is difficult to capture organoids distributed in different layers of Matrigel in a single-layer image, causing a great time consumption for researchers to gather information about the status of all organoids in the 3D culture model. Second, the assessments of organoids rely on organoid number counting and refractive index differences of organoids under bright fields, which are easily affected by researchers’ subjectivity. Third, there is still a lack of high-throughput imaging analysis methods that are easy to operate.

With the development of 3D organotypic culture models and the advances in 3D imaging systems, Z-stack technology has been widely used in the drug screening field [17–19]. Z-stack means continuous scanning of different Z-axis levels to acquire multiple images of the same field and then merge the images obtained into a high-quality image with the help of computer algorithms. In this study, by using the fluorescence dye Calcein-AM to identify living cells in organoids and using the Z-stack image processing method to capture all organoids cultured in Matrigel, a novel method was proposed that is suitable for high-throughput imaging and analyses of organoids.

## Methods

### Mice

The C57BL/6 J mice used in the experiments were purchased from Vital River (production license number: SCXK (Zhe) 2019-0001). The mice used were 8–10 weeks old and were raised in an SPF environment with a circadian rhythm of 12 h/12 h alternates. Mouse studies were approved by the Institutional Animal Care and Use Committee of Fudan University (No. 201904002Z).

### Cultivation of organoids

The intestinal organoids were developed from mouse small intestinal crypts and cultured as described by Rao et al. [20]. Briefly, mouse small intestinal fragments (about 4 cm) were isolated and washed with cold PBS with penicillin and streptomycin (15,140–122, Gibco). Crypts were collected after digesting with 5 mM EDTA (AM9260G, Ambion) and then passed through a 70-μm cell strainer to remove villi. Isolated crypts were enriched and embedded in Matrigel (356231, Corning). In this article, small intestinal crypts were planted in a 96-well plate (220400, Sorfa) in 4 μL Matrigel with 80–100 organoids per well. Small intestinal organoid culture medium was added and refreshed every 2 days.

The tumor organoids were developed from rectal cancer biopsy samples by Yao et al. [9]. In this article, four kinds of organoids, defined as chemo-sensitive or resistant to 5-fluorouracil (5-FU-s, 5-FU-r) or CPT11 (CPT11-s, CPT11-r), were selected. The chemosensitivities of tumor organoids to chemo-therapeutics agents were determined by the ratio of PDO size change at day 24 to day 0 after treatments. The mean cutoff value was 36.42% as determined by Youden’s index and bootstrap samples. Tumor organoids derived from P (v)9, P (v)55, P (v)33, and P (v)63 were reported in our previous study, and their chemo-sensitive or resistant to 5-fluorouracil (5-FU-s, 5-FU-r) or CPT11 (CPT11-s, CPT11-r) were described previously [9]. Relative details of chemosensitivities of the tumor organoids we used are shown in Additional file 1: Fig. S1. The tumor organoids were cultured at a density of 50 per 10μL Matrigel in a 48-well plate (3548, Costar). Human rectal cancer organoids (RCOs) culture medium was used and refreshed every 3 days.

### Microsphere experiments

Microspheres, a latex particle reference material (GBW(E)120021, BJHongMeng), were used to explore the appropriate step size and planting density for optimizing the Z-stack relative parameters. Microspheres of different sizes (diameter 10, 20, 30, 60, 100 μm) were resuspended in 10μL Matrigel in different densities (2–20/μL) and were planted in a 48-well plate.

### Fluorescence staining of organoids

#### Calcein-AM staining

The Calcein-AM stock was prepared by adding 10μL DMSO to 50 μg Calcein-AM powder and stored at − 20 °C according to the manufacturer instructions (40719ES50, Yeasen). The stock was diluted with PBS at a ratio of 1:1000 just prior to staining. 0.1 mM CuSO_4_ was added as needed. Briefly, when testing the ability of heavy ions to quench the nonspecific staining of Calcein-AM to Matrigel, 0.1 mM CuSO_4_ (final concentration) was added to the experimental group. When validating the chemosensitivity of tumor organoids and the radiation-mitigating effect on intestinal organoids, CuSO_4_ was also added in the dyeing buffer. Wells containing the organoids were gently washed twice with PBS and then incubated in the working solution at 37 °C. Organoids were observed after being washed with PBS.

#### Hoechst staining

The wells containing the organoids were gently washed twice with PBS prior to staining. Hoechst 33342 stock (40744ES60, Yeasen) was diluted with PBS at a ratio of 1:200 to obtain a working solution. Organoids were stained for 15–20 min and washed with PBS before observations.

#### CFDA SE staining

The CFDA SE stock solution was prepared by adding 500μL solvent to the CFDA SE dye (40714ES76, Yeasen). 1 μL of stock was diluted in 1 mL of PBS for working solution just prior to staining. After being washed with PBS, the organoids were incubated in the working solution at 37 °C for 15–20 min. The staining solution was washed away by PBS before observations.

#### PI staining

The PI working solution (40755ES64, Yeasen) was diluted 10 times with PBS according to the manufacturer instructions. After being washed with PBS, organoids were stained with PI working solution for 8 min. The staining solution was washed away by PBS before observations.

### Neutral red staining

A 1% neutral red (553-24-2, Sangon) stock solution was diluted with PBS at a ratio of 1:100 to prepare a working solution. Then, 100uL working solution was added to each well and incubated for 10 min.

### Ionizing radiation

After being seeded on the plate for 24 h, small intestinal organoids were exposed to ionizing radiation at a dose of 8 Gy (PXI RAD-320X, USA). The program parameters were as follows: 250 kV, 12 mA, SSD: 50 cm, dose rate: 190 cGy/Min, filter: 1 mm Al.

### Agents

A thymidylate synthase inhibitor 5-fluorouracil (5-FU, S1209, Selleck) and a topoisomerase 1 inhibitor irinotecan (CPT-11, S2217, Selleck) were used in chemosensitivity studies of tumor organoids. When organoids sizes reached 100 μm (about 2–3 days culture), 10 μM of 5-FU or CPT-11 was added, respectively. The surviving organoids were evaluated 10 days later.

To validate the radioprotective effects of radioprotectants, single dose of bFGF (200 ng/mL), NAM (10 mM), and SGC (5 μM) was added, respectively, at 24 h post-irradiation. The number and size of organoids were analyzed 4 days later (the fifth day post-irradiation).

### Imaging of microspheres and organoids

The organoids and microspheres were checked under a Nikon microscope (Ti2-E, Nikon), and the images were captured by NIS-Elements AR software. Extended depth of field (EDF) in ImageJ software was chosen as the algorithm to merge images from different layers into the final one when performing the Z-stack technique. The pictures were then analyzed by the GA3 analysis system, a customizable software suitable for high content data measurement provided by Nikon company. The organoids' number and size (area) were obtained by delineating the surviving organoids in bright field or fluorescent field, and the survival rates were further calculated as percentage of survival organoids to initial organoids (% of d0). The background in fluorescence staining was evaluated on incubated Matrigel without organoids. The average values of fluorescent intensity in fluorescence channel (green for Calcein-AM staining) were calculated from nine diffused points in Matrigel.

### Statistical analysis

GraphPad Prism software (version 8.0) was used for the statistical analysis. Statistical tests for normality and equal variance were performed, and the experimental data were in accord with normal distribution and uniform variance. A two-tailed Student’s t test was performed to examine the differences between the control group and the treated group (Figs. 4, 5, 7). The data are presented as the mean ± standard deviation (SD) or standard error of the mean (SEM).

## Results

### The Z-stack imaging technique could capture an accurate image of 3D organoids

**Fig. 1:**
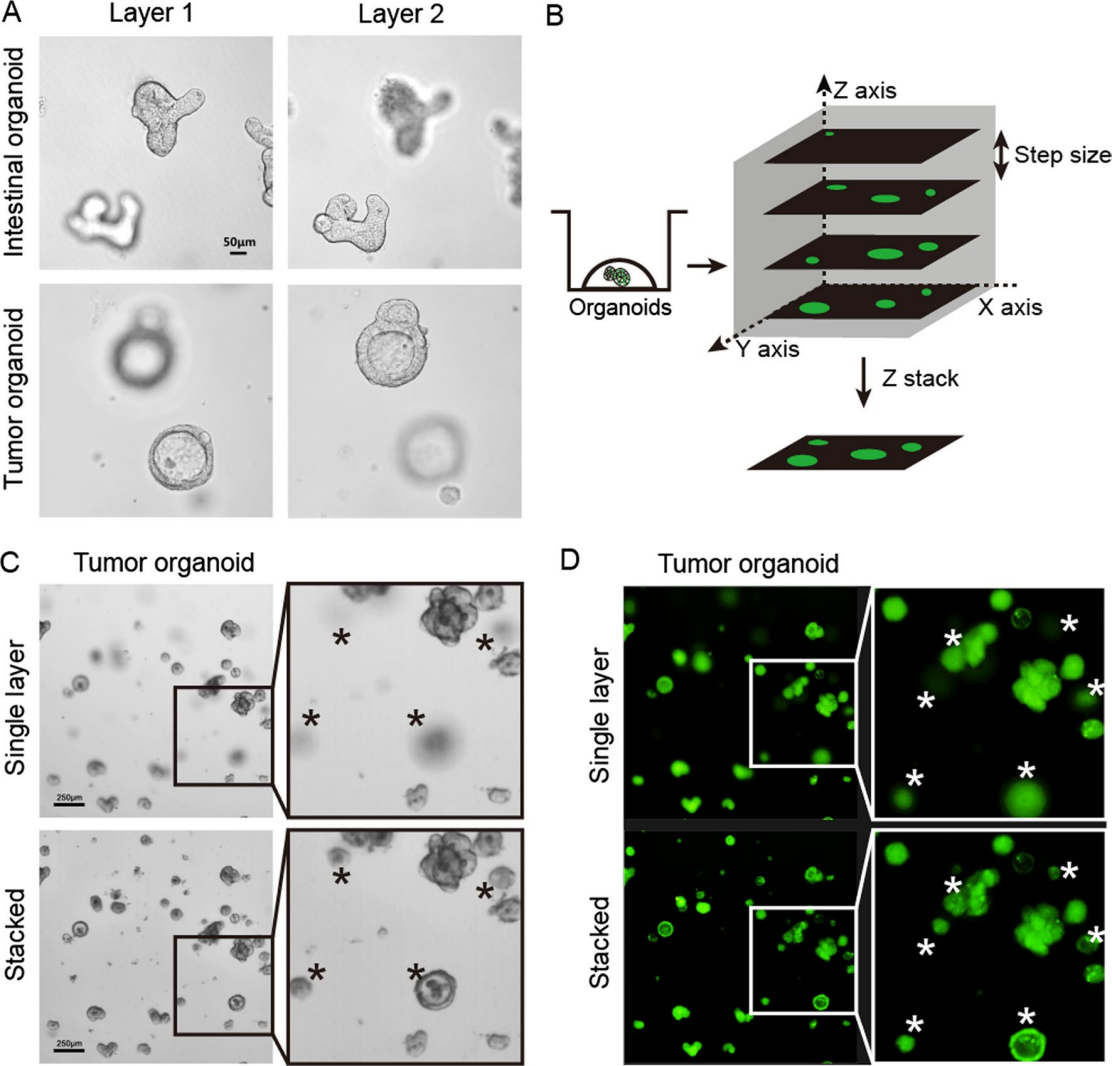
Using the Z-stack imaging technique could achieve high-quality images of 3D organoids. **A** Representative pictures in the same field show that it is difficult for researchers to catch all organoids in a single-layer image. **B** Schematic diagram of Z-stack imaging technique. **C**, **D** Images obtained by continuous screening of the same well from multiple layers were used to make a final image (**C** bright field, **D** fluorescent field). Top: Representative single-layer images. Bottom: Representative images after executing Z-stack techniques on multiple layers. *Organoids that were blurred in the single-layer photograph were clear in the Z-stacked photograph

When one focuses on organoids living in the same layer, the image of organoids distributed in other layers is often blurred (Fig. 1A). In this study, by using Z-stack technology, accurately imaging of 3D organoids cultured in different layers was attempted. In our tests, the scanning time for 2, 4, 7, 10, and 14 stacks for a 48-well plate under both bright and fluorescent field were 156 s, 320 s, 556 s, 791 s, and 1112 s, respectively. Under both bright field and fluorescent field, compared with the single-layer image, the Z-stacked image showed good performance for organoids that were blurred in the single-layer image. Organoids that were blurred in the single-layer imaging obtained a sharp border and more details in the Z-stacked image (Fig. 1C, D). Here, we presented the differences between the single-layer image and Z-stacked image (Additional file 1: Fig. S2).

### Optimization of Z-stack imaging assay

**Fig. 2:**
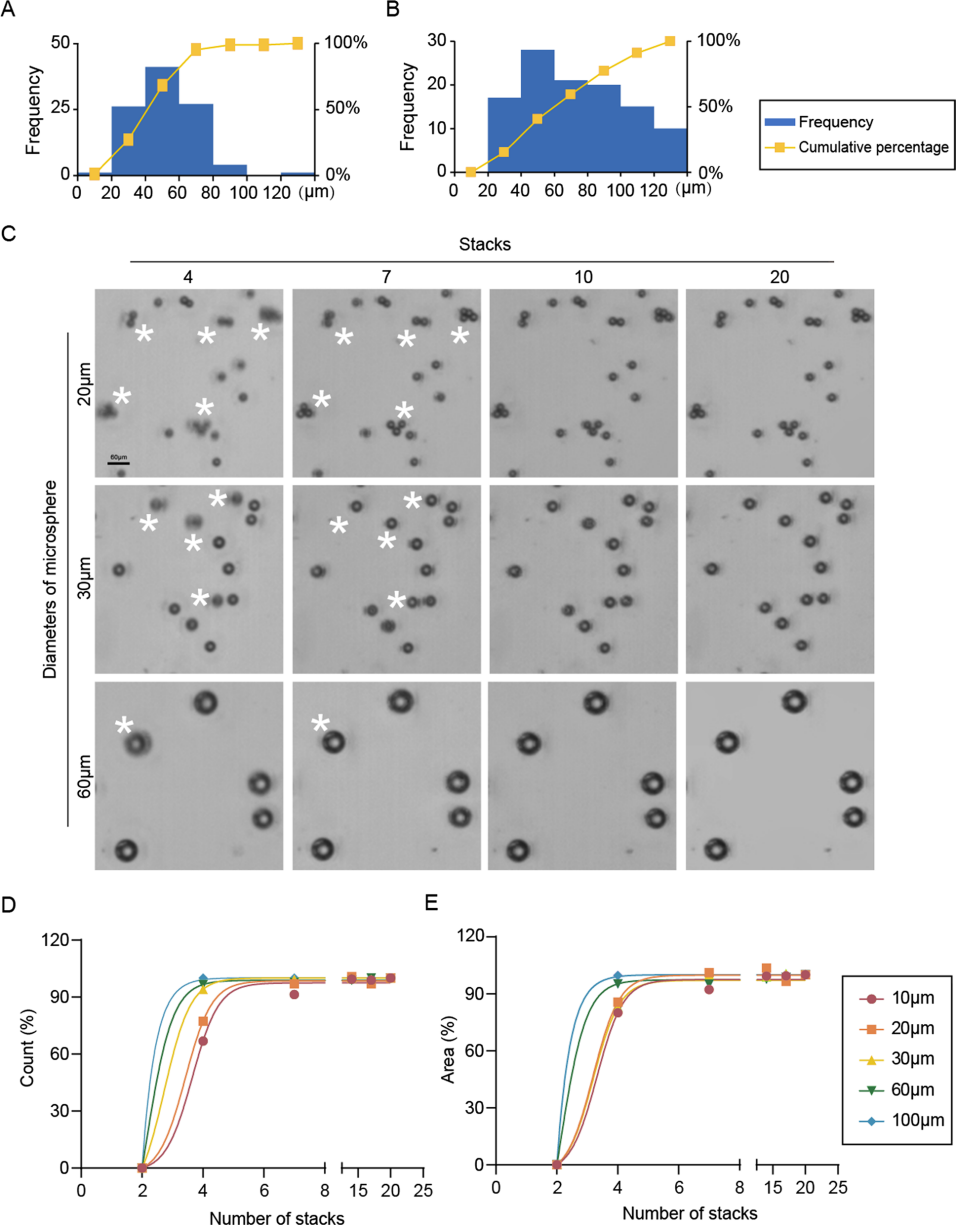
Explorations on the appropriate step size of Z-stack. **A** The size distribution of surviving tumor organoids on the tenth day after chemotherapy treatment, as determined by short diameter measurement. **B** The size distribution of regenerated intestinal organoids on the fifth day post 8 Gy irradiation, as determined by short diameter measurement. **C** Examples of imaging of microspheres of different sizes when using different numbers of stacks to screen. The range of performing Z-sack was 1100 μm. **D**, **E** The numbers and areas of microspheres varied with the different numbers of Z-stack layers. *Organoids that were blurred became clear with the increase of stacks

**Fig. 3:**
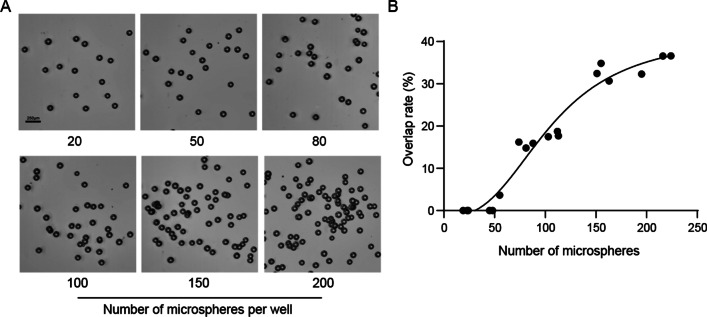
Optimization of microsphere density in 10μL Matrigel. **A** Representative images of microspheres grown at different densities. The microspheres were implanted into 10μL Matrigel in a 48-well plate. The number indicates the number of microspheres in each well. **B** The overlap rate increased as the number of microspheres increased

With the increase in the number of scanning layers, the quality of the Z-stacked image gradually increases. However, with the increase in scanning layers, scanning time would be increased. We first evaluated the size distributions of regenerated intestinal organoids at the fifth day post 8 Gy irradiation (IR), and of surviving tumor organoids at the tenth day after chemotherapy treatment. As shown in Fig. 2A, B, nearly all of the regenerated organoids were between 20 and 100 μm in short diameters, while the regenerated intestinal organoids were larger. To further explore the appropriate Z-stack step size, different sizes of microspheres (10–100 μm in diameters) were used in a 10μL Matrigel culture. The distance between the top and bottom of the Matrigel was 1100 μm. As the results showed, both the number and the size of the microspheres gradually increased before they entered a plateau with the increase of scan layers, and this was especially obvious in the imaging of small microspheres (Fig. 2). Taking 20 μm microspheres as an example, when the scan layers increased to 7 (the corresponding step size was 180 μm), 95% microspheres could be captured. A further increase in the layer number did not bring an obvious improvement in the imaging quality, and both the number and total area of microspheres stabilized at a certain level. For microspheres with larger sizes under the same parameter settings, it was found that with the increase of the size of microspheres, the counting and total cross-sectional area of microspheres reached a plateau earlier. When imaging microspheres with diameters of 60 μm and 100 μm, there was no obvious rising phase before reaching the plateau stage. This was because even four layers were enough to capture a clear image for microspheres under these sizes (Fig. 2). Based on these experimental results, considering the scanning time and the surviving organoids’ sizes after treatments, we concluded that 180 μm is a suitable step size for Z-axis layer scanning. At this step size, small-sized 3D biological samples would not be missed during imaging. Furthermore, we used 100 μm standard microspheres to explore an appropriate planting density of organoids. As the results showed, with the increase in organoid density, the overlap rate of organoids gradually increased. The range of 20–50 was an appropriate density for organoid planting in 10 μL Matrigel culture (Fig. 3).

### The fluorescence dye Calcein-AM could effectively identify living cells in organoids

**Fig. 4:**
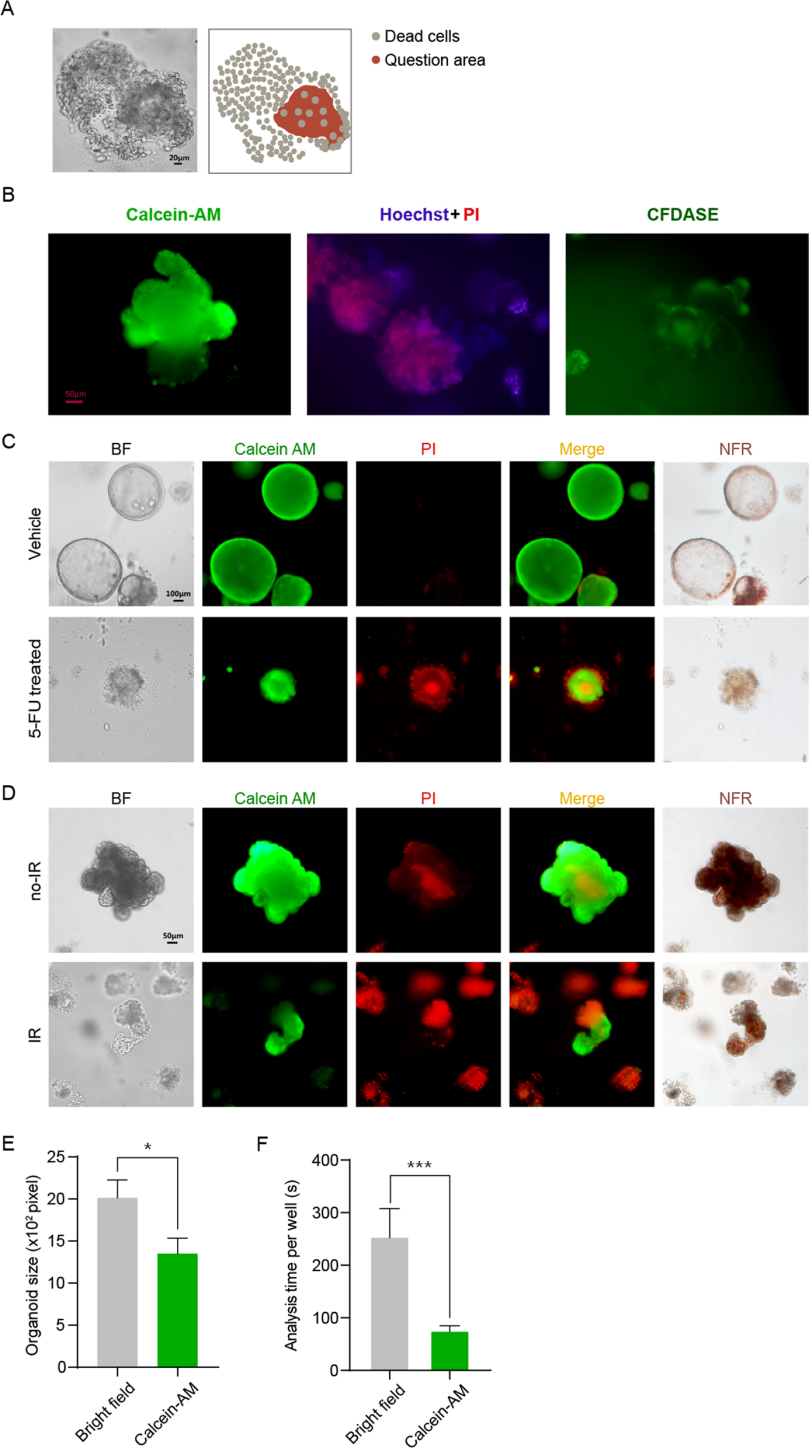
Fluorescence dye could effectively identify living or dead cells in organoids. **A** Representative image of an illegible tumor organoid for researchers to identify under bright fields. After being treated with 5-FU, the organoid was composed of loose peripheral dead cells and an area with unsure living or dead cells. **B** Comparison of the staining effects of different fluorescent dyes. **C** Calcein-AM and PI can effectively mark living or dead cells in patient-derived tumor organoids before and after treatment. **D** Calcein-AM and PI identified living or dead cells in mouse small intestinal organoids. Neutral red staining was performed at the same time, and the results were consistent with Calcein-AM staining. **E** Comparison of the cross-sectional areas of organoids under bright field and fluorescent field on the fifth day after 8 Gy irradiation. Data are presented as the mean $\pm$ SEM, n = 39. **F** Comparison of the time required to delineate the areas of the surviving organoids under the bright field and fluorescent field, n = 5. Data are presented as the mean $\pm$ SD, n = 5. *P < 0.05, ***P < 0.001

In contrast to cells, organoids have a three-dimensional multicellular structure. After being treated with drugs or IR, the structure of an organoid becomes loose and illegible. The dead cells would be scattered in the original organoid growth position and cover the living and regenerating cells, making it difficult for researchers to assess the status of the organoids efficiently and accurately (Fig. 4A). Therefore, three different fluorescence marking methods, Calcein-AM, Hoechst/PI, and CFDA SE, were compared to find an appropriate dye for organoid staining (Fig. 4B, Additional file 1: Fig. S3). As shown in Fig. 4B, compared with the other staining methods, Calcein-AM staining had a lower background intensity and a sharper outline of organoids. We also compared the three different fluorescence marking methods under different concentrations, and the results showed consistency (Additional file 1: Fig. S3). Thus, Calcein-AM was chosen as the appropriate fluorescent dye for organoids. Subsequently, we performed Calcein-Am/PI double staining on tumor organoids before and after drug treatment, and on mouse small intestinal organoids before and after IR. Neutral red staining was performed at the same time to assist in assessing the status of organoids. As shown in Fig. 4C, D, the living cells in the organoids could be well stained by the green fluorescent dye Calcein-AM, while the surrounding dead cells were stained by the red fluorescent dye PI. Calcein-AM and neutral red showed the same staining area. Compared with untreated organoids, treated organoids exhibited a greater proportion of red fluorescence area. The delineated areas of surviving organoids on the fifth day after 8 Gy IR under bright fields and fluorescence fields was also compared. As shown in Fig. 4E, the delineated area of surviving organoids under the fluorescent field was smaller than that of the bright field. It was speculated that this was because that part of the surviving cells was surrounded by dead cells, resulting in an inaccurate delineated area in the bright field, while this phenomenon could be avoided under the fluorescent field. Compared with delineation in the bright field, the fluorescent area sketch was highly efficient and time-saving (Fig. 4F).

### Optimization of Calcein-AM staining

**Fig. 5:**
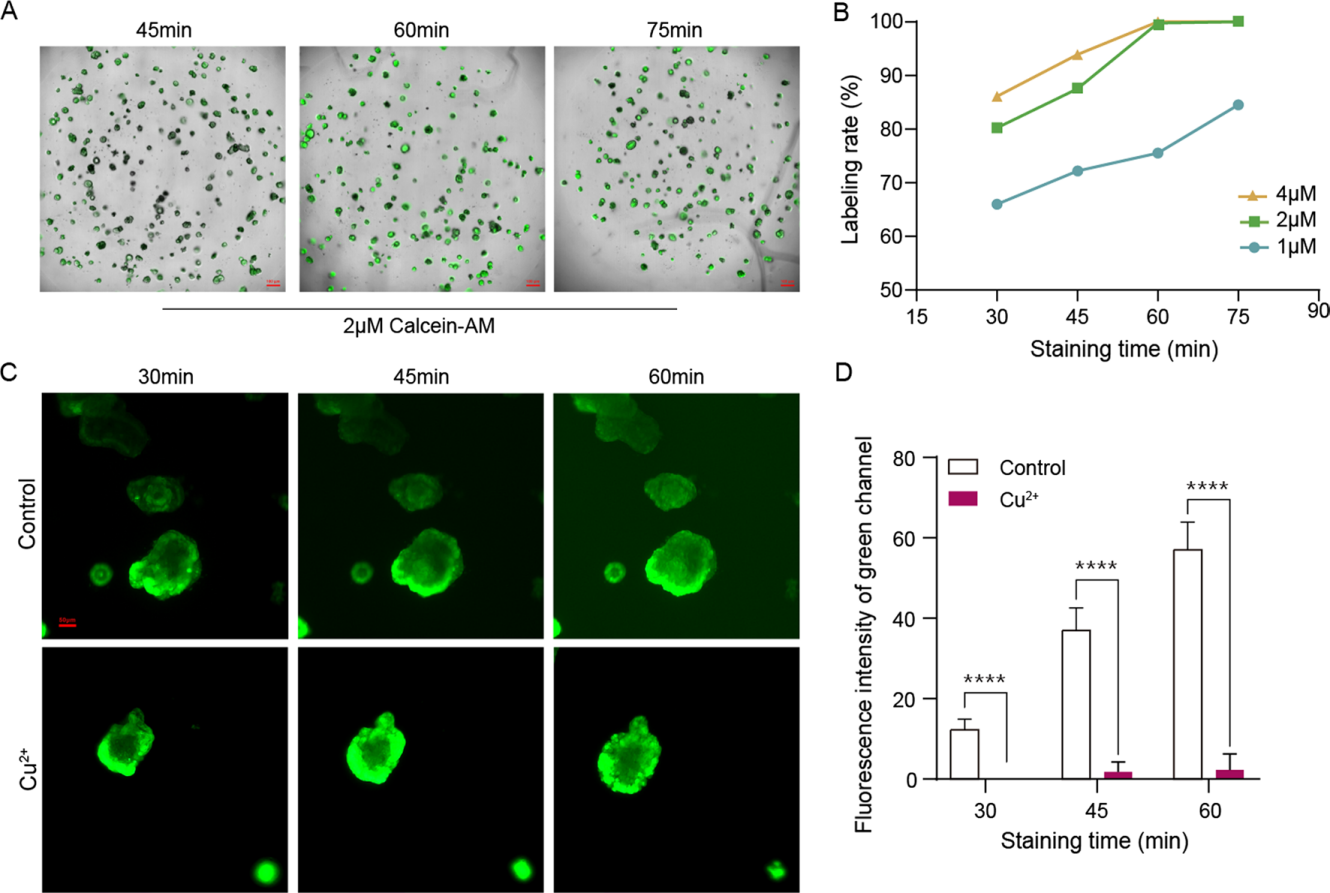
Optimization of Calcein-AM fluorescent staining method.** A**, **B** Variation of tumor organoid labeling rates with different staining times and dye concentrations. **C**, **D** 0.1 mM Cu^2+^ effectively reduced the staining background during the staining process of mouse small intestinal organoids. Three wells were measured for each group and data of 27 points were obtained. ****P < 0.0001

When performing fluorescent staining on organoids, the staining time and dye concentration are critical. Therefore, the effects of different staining times and dye concentrations on staining efficiency were explored. Approximately 150–200 tumor organoids were plated in each well within 10 μL Matrigel. As shown in Fig. 5A, B, at the same concentration, as the staining time increased, the labeling rate of organoids gradually increased. For the same staining time, a higher dye concentration paralleled with a higher organoid labeling rate. It was noticed that when the organoids were stained with 2 μM Calcein-AM for 60 min, the marking rate reached 100%. Subsequent experiments were also implemented around these parameters. During the experiments, it was found that as the staining time increased, the green fluorescent background of Matrigel would gradually deepened. Heavy ions to quench the nonspecific staining of Calcein-AM to Matrigel were attempted. As shown in Fig. 5C, D, 30 min after staining, nonspecific staining of Matrigel was observed in the control group, and the fluorescence intensity of the green channel gradually increased with the extension of staining time. The results showed that the addition of 0.1 mM Cu^2+^ could effectively reduce the fluorescence intensity of Matrigel over a long period of staining, which allowed researchers to perform accurate analysis of intracellular fluorescence.

### Validation of a high-throughput organoid imaging assay by testing the chemosensitivity of tumor organoids

**Fig. 6:**
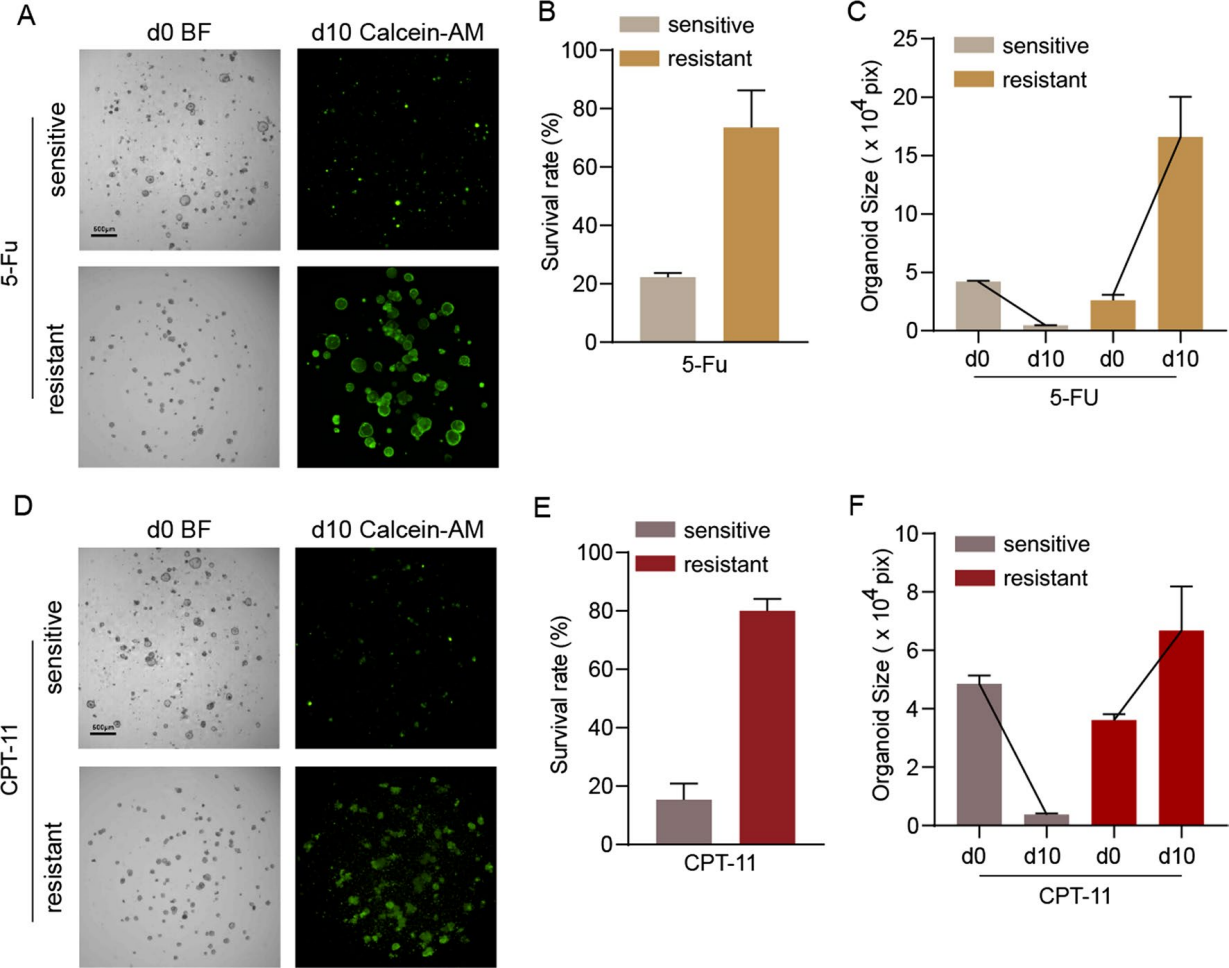
Validation of a high-throughput organoid imaging assay by testing the chemosensitivity of tumor organoids. **A** Presentation of organoids that are sensitive or resistant to 5-FU (10 μM). D0 refers to the day of drug treatment and d10 means the tenth days post-d0. **B**, **C** The survival rate and total area of tumor organoids on day 10 after 5-FU treatment. The d0 count was set as the standard. A total of 176 organoids were measured in the 5-FU-sensitive group and 150 organoids were measured in the 5-FU-resistant group. **D** Presentation of organoids that are sensitive or resistant to CPT11 (10 μM). **E**, **F** The survival rate and total area of tumor organoids on day 10 after CPT-11 treatment. A total of 188 organoids were measured in the CPT-11-sensitive group, and 180 organoids were measured in the CPT-11-resistant group. Data are presented as the mean $\pm$ SD

Previous work showed that the size of PDOs could be a reliable parameter to predict the response of locally advanced rectal cancer to chemoradiation treatment [9]. However, the process of outlining the surviving area of organoids under bright fields was time-consuming. Therefore, an attempt was made to use the Z-stack technique and fluorescence labeling method to establish a quick and efficient method for the analysis of the chemosensitivity of tumor organoids. The statuses of the organoids at the initiation of the chemotherapy treatment were captured under a bright field, and the organoids’ sizes on the tenth day after the chemotherapy treatment were captured by a combination of the Z-stack technique and fluorescence labeling methods. Organoids that were sensitive and resistant to 5-FU and CPT-11 were used to explore whether the fluorescent area of organoids could replace the cross-sectional area of organoids drawn manually in bright fields. As shown in Fig. 6A, B, the fluorescent dye Calcein-AM could accurately label living cells in organoids, and the area of surviving organoids that were resistant to 5-FU or CPT-11 could be clearly displayed under the fluorescence field. Compared with the resistant group, there was a significant decrease in the fluorescent areas of sensitive organoids (Fig. 6). We also performed the CTG assay for benchmarking. As the results showed, the CTG curve and fluorescence cross-sectional area curve showed the same trend without significant differences (Additional file 1: Fig. S4).

### Validation of a high-throughput organoid imaging assay by testing the radiation-mitigating effect on intestinal organoids

**Fig. 7:**
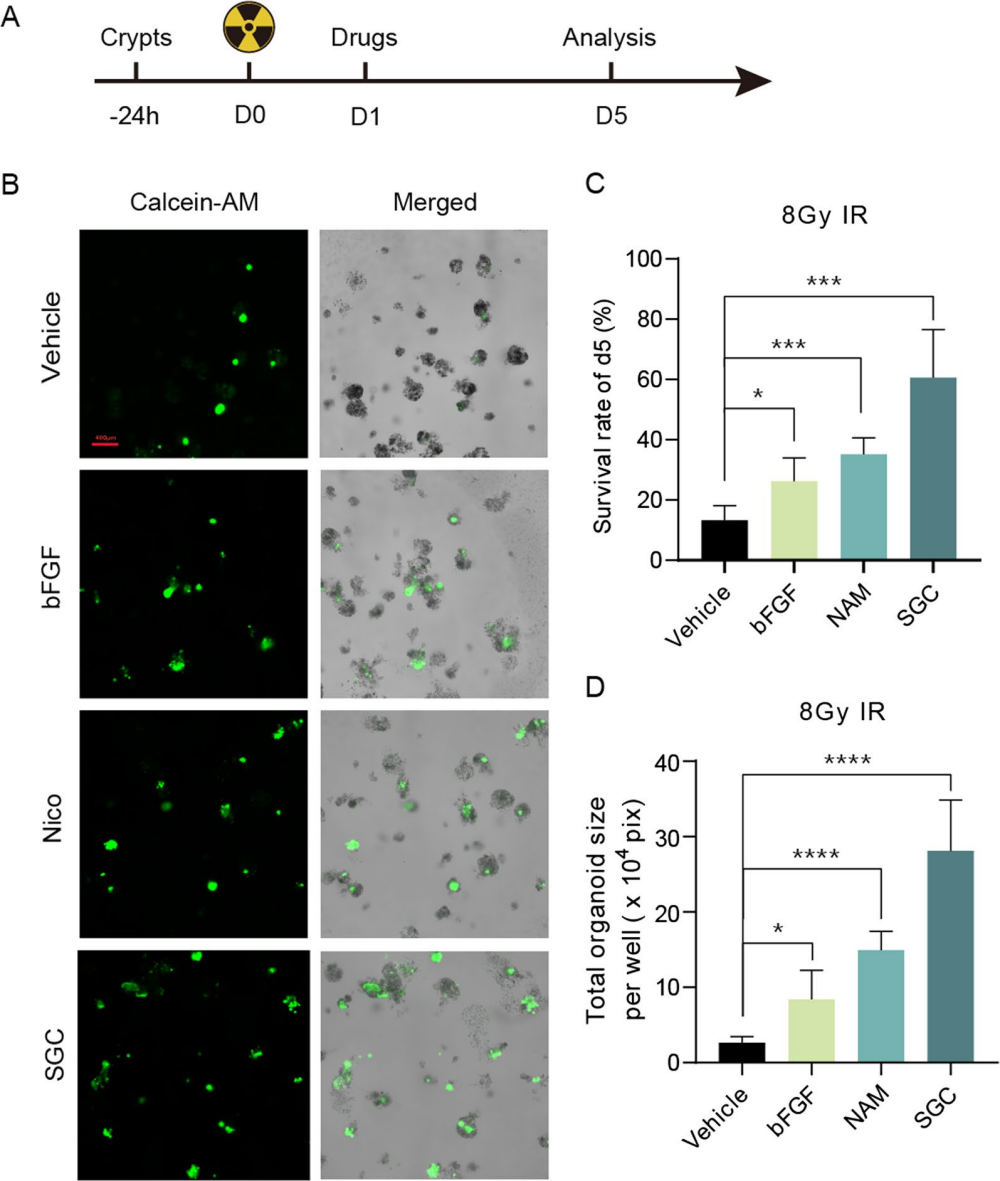
Validation of a high-throughput organoid imaging assay by testing the radiation-mitigating effect on intestinal organoids. **A** Workflow for verifying the radiation-mitigating effects of drugs on mouse intestinal organoids after 8 Gy irradiation. **B** The radiation-mitigating effects of bFGF, NAM, SGC on small intestinal organoids at d5 after 8 Gy irradiation. **C**, **D** Compared with the vehicle group, the bFGF (200 ng/mL), NAM (10 mM), and SGC (5 μM) groups had significantly higher survival rates of small intestinal organoids, and the total organoids sizes per well were larger. 57, 82, 145, and 241 surviving organoids from five images were analyzed in the Vehicle, bFGF, NAM, and SGC group, respectively. Data are presented as the mean $\pm$ SD (n = 5). *P < 0.05, ***P < 0.001, ****P < 0.0001

Additionally, an attempt was made to use this organoid platform to verify the radioprotective effects of three radiomitigators that have been reported. The workflow of the experiment is shown in Fig. 7A. The count and area of surviving organoids were measured on the fifth day after 8 Gy IR. Compared with the control group, the bFGF group [21, 22], NAM group [23, 24], and SGC group [20] had significantly more surviving organoids and larger fluorescent areas. Among the three radioprotective agents, SGC showed the strongest radioprotective effect, which was consistent with the previous work (Fig. 7B–D).

## Discussion

Wenzel and colleagues’ work showed the superiority of the 3D culture model in the drug screening field because 3D models have a complex multicellular structure and could identify compounds that would not be screened out by 2D cell lines [25]. Kim et al. compared multiple parameters such as living cell counts, organoid surface area, and organoid volume to analyze organoid growth and drug response. Their work showed that in addition to the volume of organoids, the cross-sectional area of organoids was also a reliable parameter to measure the growth of organoids [26]. Here, we proposed using the Z-stack imaging technique to gather all organoids’ maximum cross sections in one image. Compared with traditional imaging techniques, Z-stack technology can obtain clearer organoid images. We further optimize the relevant parameters of the Z-stack. Using different sizes of microspheres, we concluded that 180 μm (7 scanning layers) is an appropriate step distance for Z-stack image. When the stack number reached 7, 10, and 14, the objectives in the stacked images showed sharper outline, which is sufficient for the accuracy requirement of the following analysis. As 7 is the minimal essential stack number to improve accuracy and overcome the intractableness for the following analysis, we chose 7 stacks in our study.

The dead cells in organoids make it difficult for researchers to assess the status of organoids. This study showed that the green fluorescence dye Calcein-AM could specifically mark living cells in organoids with high efficiency, which is also suitable for organoids after compound treatment. Based on this result, we further explored the appropriate timing and concentration for Calcein-AM staining. Our work shows that 2 μM and 60 min are suitable parameters for organoid staining. We also found that Cu^2+^ quenched the nonspecific staining of Matrigel by Calcein-AM.

The traditional CTG method used to evaluate the vitality of organoids has excessive variability within groups, and the organoids used for CTG analysis could not continue to be observed [9]. Chen et al. reported a great deep learning-based method to evaluate the invasiveness of 3D tumor organoids under bright fields [27]. Lee et al. established an automated image analysis system to assess the aggregation conditions of organoids. Using this method, they screened concentration combinations of Matrigel and growth factors for analyzing their impact on the organoid formation [28]. Bulin et al. used the Calcein-AM/PI double staining method and CALYPSO image analysis procedure to analyze various cancer treatment effects on organoids [29]. Bode et al. used the ratio of PI/Hoechst signals to calculate cell death in organoids [30]. Here, we used the Z-stack technology to obtain the maximum cross-sectional area of organoids and optimized the parameters of the Z-stack imaging method for organoids. We used our platform to test the chemotherapy effects of 5-FU and CPT-11 on different tumor organoids. The results showed that using our method could distinguish the different sensitivities of organoids to drugs. We also used this method to verify the radioprotective effects of radioprotectants. The results showed that our system could effectively identify the protective effect and realize the drug screening. Compared with the organoid drug screening platforms reported thus far [27–33], our platform has lower equipment requirements, improved accuracy of organoid imaging and is easy to handle. Besides, organoids labeled by low concentration Calcein-AM can still be used for follow-up observations.

In summary, firstly, we established the Z-stack imaging assay by focusing on the maximum cross-sectional area and optimized parameters including the number of scanning layers and the step sizes of scanning. The optimizations saved plenty of scanning time without sacrificing the quality of images. Secondly, we combined Z-stack imaging technology and fluorescence labeling method to image and assess 3D organoids. This imaging and analysis workflow kept the integrity and morphological information of organoids in the course of processing. Finally, we used the organoid size ratio parameter to verify the chemosensitivity of tumor organoids and the radioprotective effects of agents on small intestinal organoids. By using this organoid size ratio method, we could assess the efficiency of radioprotective agents for organoids on the fifth day after radiation, showing application potential for pharmaceutical companies. Compared with the traditional manual evaluation method, the organoid size ratio method is more objective, rapid, labor-saving, easy to operate, and suitable for high-throughput drug screening.

## Conclusion

In summary, we established the Z-stack imaging assay by focusing on the maximum cross-sectional area and optimized the organoid size ratio method to verify the influences of different drugs on 3D organoids. This method is suitable for high-throughput drug screening and should facilitate the applications of organoids in drug development and precision medicine.

## Supplementary Information

**Additional file 1**.** Fig. S1**. The chemosensitivities of 4 tumor organoids used for validation of the high-throughput organoid imaging assay.** Fig. S2**. The comparison of the singlelayer and Z-stacked image.** Fig. S3**. The comparison of three staining methods under different concentrations.** Fig. S4**. Comparing CTG methodand fluorescence labeling method to test the chemosensitivity of tumor organoids.

## Data Availability

The data are available from the corresponding author on reasonable request.

## Abbreviations

2D: Two-dimensional
3D: Three-dimensional
PDOs: Patient-derived organoids
EDF: Extended depth of field
MIP: Maximum intensity projection
AIP: Average intensity projection
IR: Irradiation
Gy: Gray
PBS: Phosphate-buffered saline solution
PI: Propidium iodide
5-FU: 5-Fluorouracil
CPT-11: Irinotecan
NAM: Nicotinamide
bFGF: Basic fibroblast growth factor
SGC: SGC-CBP30
