# Supplementary material for: Assay establishment and validation of a high-throughput organoid-based drug screening platform
Source: Stem Cell Res Ther. 2022 May 26;13:219. doi: 10.1186/s13287-022-02902-3 (PMC9137096; doi:10.1186/s13287-022-02902-3)
Supplement: Supplementary file 1 — Additional file 1. Fig. S1. The chemosensitivities of 4 tumor organoids used for validation of the high-throughput organoid imaging assay. Fig. S2. The comparison of the singlelayer and Z-stacked image. Fig. S3. The comparison of three staining methods under different concentrations. Fig. S4. Comparing CTG methodand fluorescence labeling method to test the chemosensitivity of tumor organoids. [file 13287_2022_2902_MOESM1_ESM.pdf]

Additional file 1 for

Assay establishment and validation of a high-throughput organoid-based drug screening platform

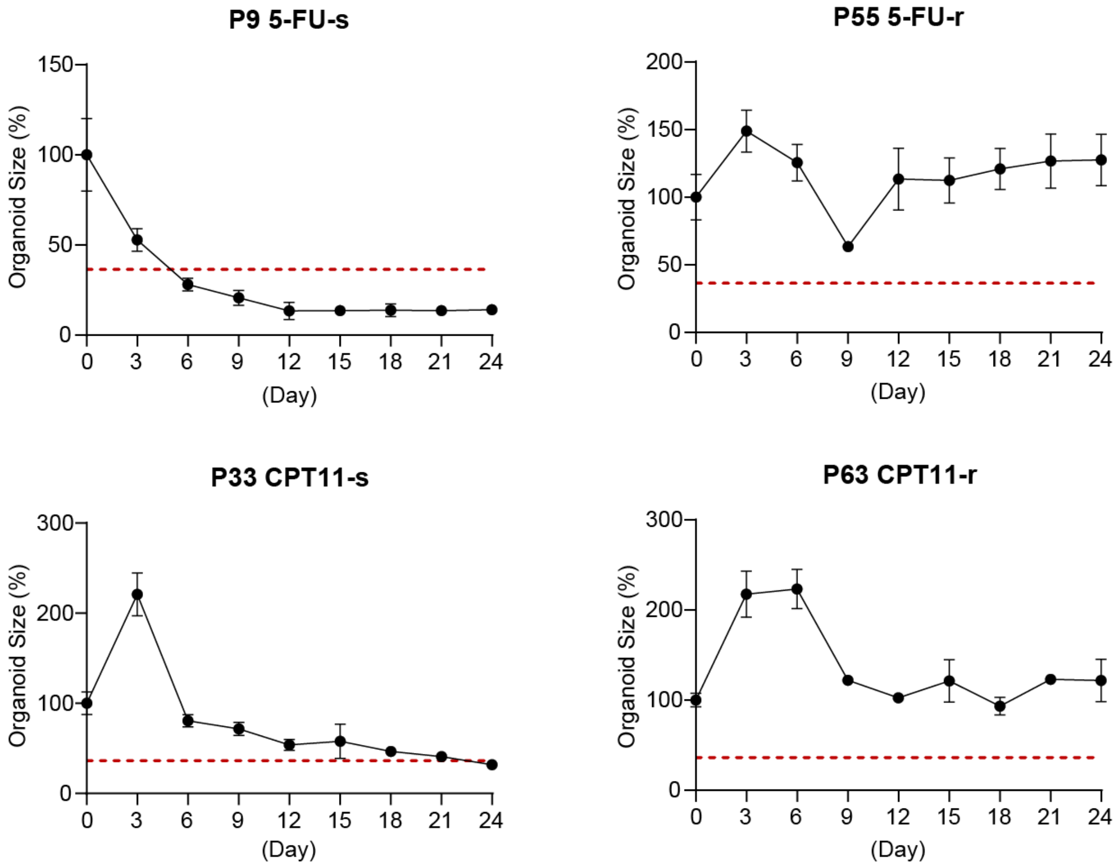

Additional file 1: Fig. S1

The chemosensitivities of 4 tumor organoids used for validation of the high-throughput organoid imaging assay. Organoid size changes after being treated by 10μM 5-FU or 10μM CPT11 in 4 organoid lines. 5-FU-s, 5-FU-r: Organoids defined as chemo sensitive or resistant to 5-Fluorouracil; CPT11-s, CPT11-r: Organoids defined as chemo sensitive or resistant to CPT11. Organoid size data shown are mean ± SEM, n=3.

A

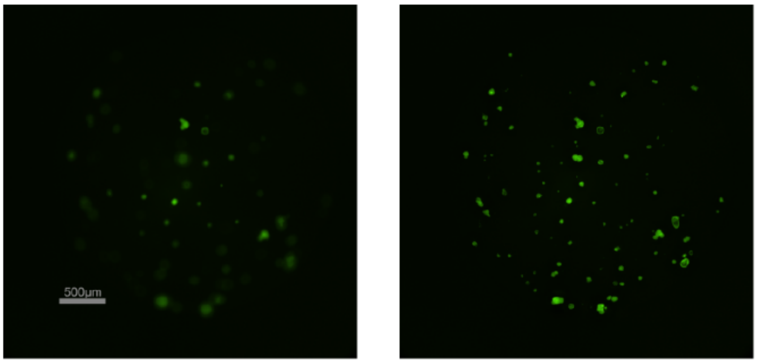

Single-layer Z-stacked

B

Comparison of the single-layer image and Z-stacked image

Single layer		Z-stacked	
Count	Total Area (pixel)	Count	Total Area (pixel)
8	8787	40	22521

Additional file 1: Fig. S2

The comparison of the single-layer and Z-stacked image.

A. Representative single-layer image and Z-stacked image under the same field; **B.** Comparison of the single-layer and Z-stacked image (organoids that were blurred in the image were not measured).

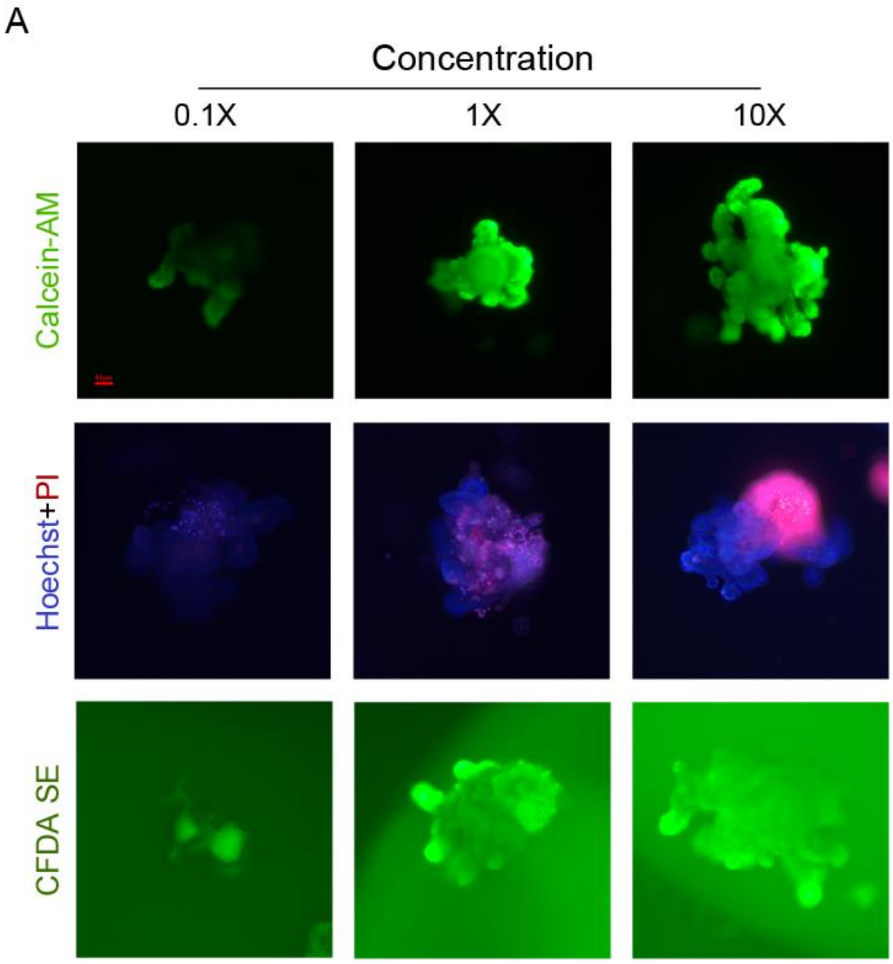

Additional file 1: Fig. S3

The comparison of three staining methods under different concentrations.

A, Calcein-AM, Hoechst/PI, and CFDA SE of different concentrations were used to stain mice small intestinal organoids. For Calcein-AM dye, “1X” concentration means 2μM, and for Hoechst/PI and CFDA SE staining, “1X” concentration means the recommended working concentration according to the manufacturers.

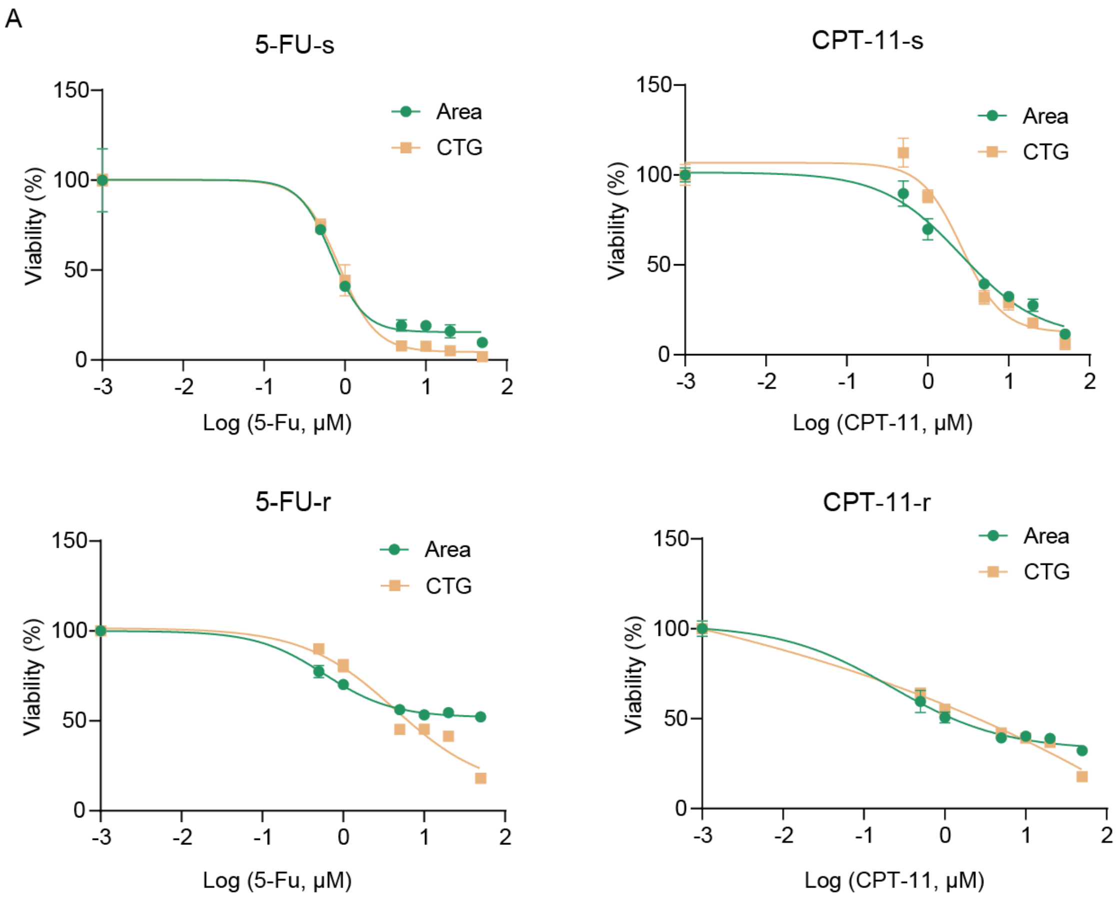

Additional file 1: Fig. S4

Comparing CTG method and fluorescence labeling method to test the chemosensitivity of tumor organoids. Cell viability changes and organoid maximum cross-section changes of organoids treated by 5-FU or CPT11. Untreated organoids were used as the starting point for both methods. Data are presented as the mean ± SD, n=3.
